# Nano tin oxide/dimethyl polysiloxane reinforced composite as a flexible radiation protecting material

**DOI:** 10.1038/s41598-023-27464-z

**Published:** 2023-01-05

**Authors:** Mona M. Gouda, Mahmoud I. Abbas, Sabbah I. Hammoury, Kareman Zard, Ahmed M.El-Khatib

**Affiliations:** 1 Physics Department, Faculty of Science, Alexandria University, Alexandria, 21511 Egypt; 2 Head of Medical Physics and Radiotherapy Department, Alexandria Ayadi Almostakbal Oncology Hospital, Alexandria, Egypt

**Keywords:** Nanoscience and technology, Physics, Health care

## Abstract

Reinforced polymer composites are a recent type of advanced shielding material that has been studied experimentally and theoretically. This work described the protection properties of silicon rubber filled with nano and micro tin oxide (II). These shielding materials are evaluated by parameters such as mass attenuation coefficient, linear attenuation coefficient, mean free path, effective atomic number, and buildup factor. The morphology and mechanical properties of silicon rubber, which is reinforced with tin oxide (II) particles in terms of weight fraction and size, have been studied. The results explain that the mass attenuation coefficient increases as tin oxide (II) concentration increases at a particular photon energy. It was found that the shielding properties of nano tin oxide (II) composites are more effective than micro tin oxide (II) composites against gamma rays. The effective atomic number values increase by increasing tin oxide (II) and so on equivalent atomic number. On the other hand, increasing tin oxide (II) weight fraction led to an increase in buildup factor maximum, which proved that tin oxide (II) concentration has significant effectiveness in radiation protection.

## Introduction

The steady increase in the use of radiation sources in many fields of our daily life demands a smart shielding material to protect workers against the hazard of radiation^1^. Radiation workers are interested in better gamma-ray protection materials and garments, that can be utilized in the hostile environment of gamma-ray exposure. Radiation therapy is a common protocol used in cancer treatment like chemotherapy and hormonal therapy. The radiation used in radiotherapy is a high energy beam (up to 21 MeV) that is used to control and demolish malignant growth^2,3^. Radiation is also used in the treatment of non-malignant tumors.

The common protective material is made from lead, which has good attenuating abilities for gamma rays. The radiation scale between (40–88) keV is absorbed by lead-based shielding garments, which have been widely used in radiation shielding to protect workers and patients in diagnostic X-rays, while this region is the regular energy scale for X-rays used in medical diagnostics. Another notable shortcoming of the composites consisting of lead is their low mechanical strength and toxicity^4,5^. So, the literature in this field has been interested in the design of new materials with suitable properties that may be utilized as shielding materials with less toxicity. Also, they have some properties that should be considered: stable material after long exposure to radiation, heat resistance, low cost, high density, high melting point, ability to mold, mechanical strength, high attenuation coefficient, low mean free path, and low half value layer.

polymers have very low density, low melting point, and low heat resistance^6^. So, polymers reinforced by another element or alloy enhance the protective properties of the final composite^7,8^. In order to select and design the reinforced material, it is important to know the type of radiation (photons in this work) and the interactions of this radiation with materials. These interactions are the photoelectric effect, Compton scattering, and pair production in the case of photons, and their probabilities of interactions change dramatically with photon energy^9–11^. Hence, the radiation attenuation properties of lead-free garments depend on beam energy and the radiation parameters at various beam qualities. Many studies discuss the performance of lead-free shielding composites in gamma rays^12^. Rammah et al*.*^13^*,* studied the radiation protection properties of silicate glasses reinforced with tin (II) oxide. They explain that shielding characteristics were improved with an increasing weight percentage of tin (II) oxide in samples. Alavian et al.^14^ discussed the shielding properties of light-density polyethylene (LDPE) filled with W of different sizes and weight fractions. They found that the weight fraction of W has a higher effect on attenuation properties than the effect of W size scale. Elsafi et al*.*^15^*,* explained the effect of iron ferrosilicon on the shielding properties of bentonite clay. The results show that powder iron has a greater effect on attenuation properties than ferrosilicon. Elkhatib et al.^16^ studied the shielding parameters of two kinds of clay. They found that the shielding properties of the mixture had been improved compared to any one of them individually.

The main cause of these demands is the direct and delayed effects of radiation exposure on workers’ and patients’ tissues. Where the exposure results in a mutation in the living cells according to the radiation absorbed dose. So, the present work aims to study the potential for the protection of silicon rubber filled with nano and micro tin oxide (II) and their mechanical properties and morphology, which can enhance radiation protection quality.

## Fabrication of SR/SnO_2_ composites

**Figure 1:**
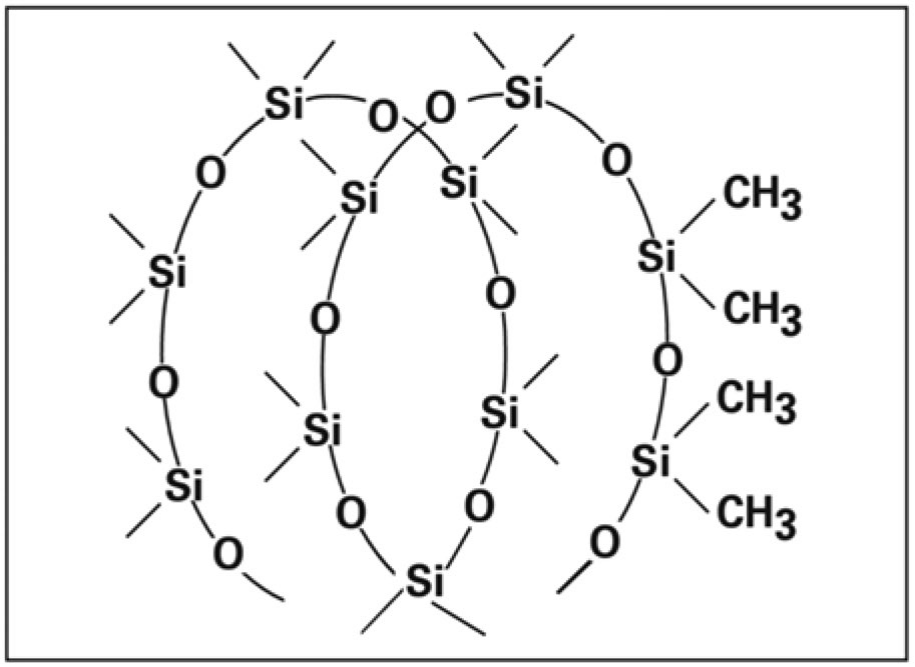
The structure of Dimethyl polysiloxane “Silicon Rubber”.

Silicon rubber is a thermoset polymer that can’t be remolded and is preferred as a radiation protection material filler with a high Z material^17^. The backbone “main chain” of silicon rubber (dimethyl polysiloxane) is formed of the siloxane bonds (–Si–O–Si–) as shown in Fig. 1 They are highly stable^18^. The filler is used to enhance the attenuation parameters of the composites. Different fillers as well as different polymers were used to prepare a reinforced polymer composite. Some studies have been conducted to investigate the nano- and micro-particle reinforced composites to be used as shield against gamma ray and neutron flux^19,20^. In this study, we used tin oxide (SnO_2_) as a filler in nano and micro sizes. The nano-tin oxide was supplied by Nanotech Co, (Egypt). Free silicon rubber, micro and nano composites with 20 and 50% tin oxide filler weight concentration were prepared by the mixing process for 15 min to get a homogeneous mixture. The mixture was cured by a vulcanizing agent. The mixture was poured into the mold at room temperature.

## Characterization of surface morphology

**Figure 2:**
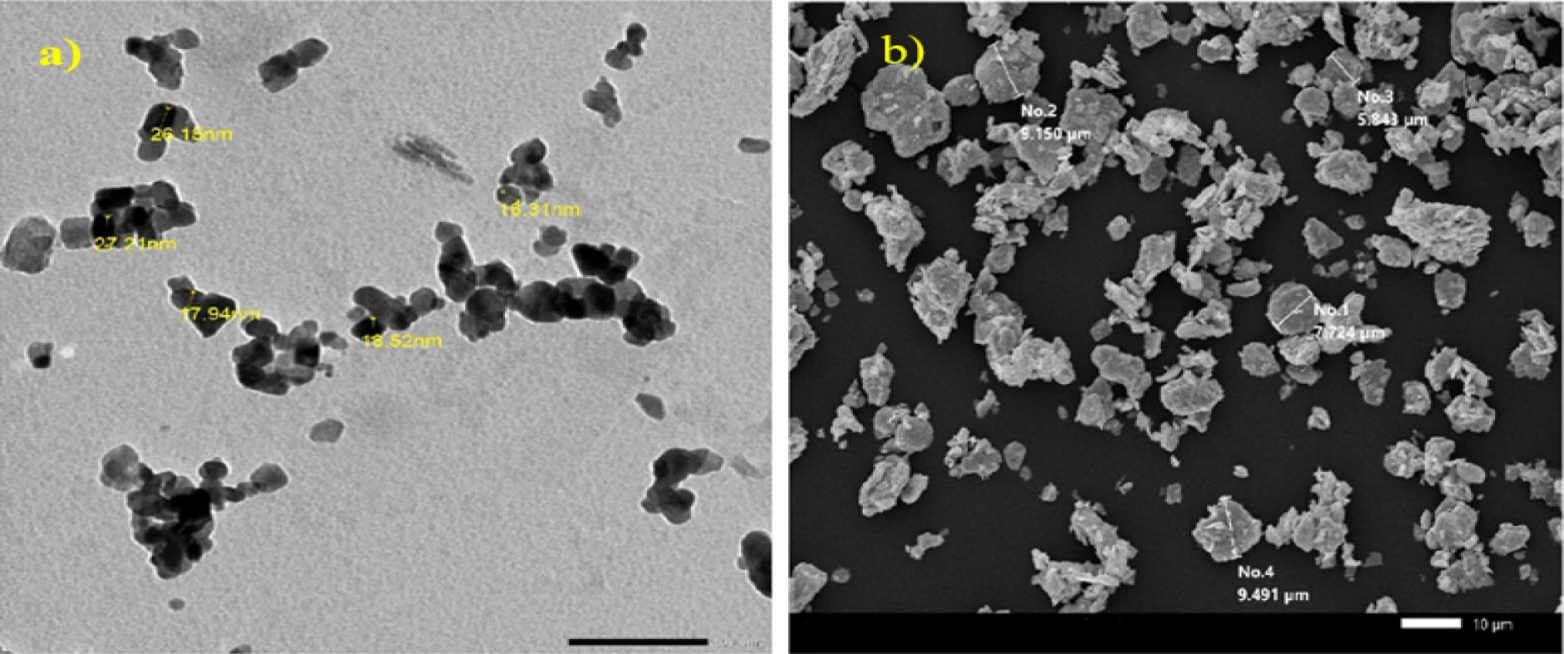
(**a**) TEM image of nano-Tin oxide particles, and (**b**) SEM image of micro-Tin oxide.

The particle size of nano tin oxide and micro tin oxide was measured by a Transmission Electron Microscope (TEM) [JEM-2100F, JEOL, Japan] at 200 kV as shown in Fig. 2. The average size of nano tin oxide was 19 nm with a standard deviation of 4.79 nm, while the average size of micro tin oxide was 9 μm.

Scanning Electron Microscope (SEM) [JSM-6010LV, JEOL] imaging has been checked to investigate the dispersion of micro and nano particles in the composites for all samples. Scanning images of composites were evaluated to determine the difference between samples. In this way, (SEM) produced an image by scanning the sample with a high-energy electron beam. While the electrons interact with the sample, they produce backscattered electrons, secondary electrons, and characteristic X-rays^21,22^. These signals are collected by detectors to form images where many signals are produced as a result of interaction inside the sample. When a beam of electrons hits the sample surface, it penetrates the sample to a depth of a few microns, which depends on sample density and accelerating voltage.

**Figure 3:**
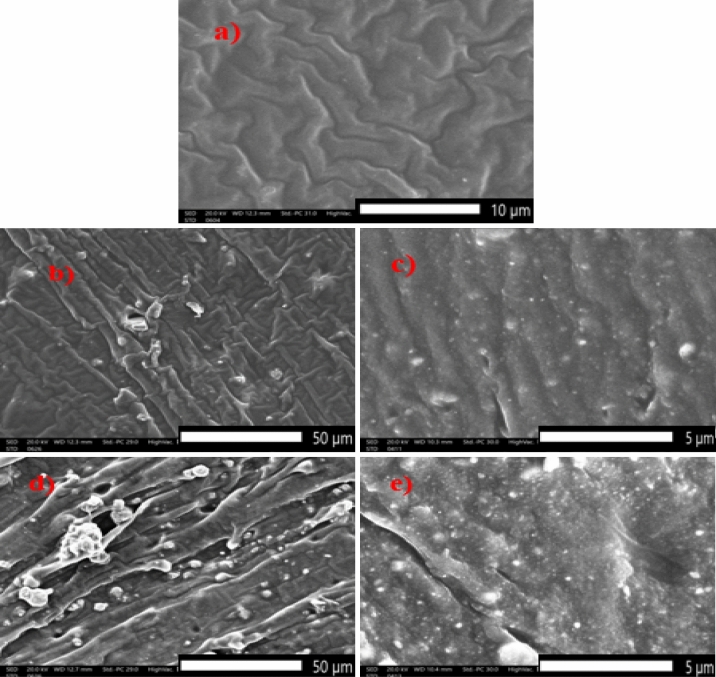
SEM image of (**a**) free silicon rubber, (**b**) 20% micro tin oxide/silicon rubber, (**c**) 20% nano tin oxide/silicon rubber, (**d**) 50% micro tin oxide/silicon rubber, and (**e**) 50% nano tin oxide/silicon rubber.

The scanning electron microscope was used to characterize free silicon rubber and tin oxide/silicon rubber composites. Figure 3 illustrates the SEM of free silicon rubber, 20% micro tin oxide/silicon rubber, 20% nano tin oxide/silicon rubber, 50% micro tin oxide/silicon rubber, and 50% nano tin oxide/silicon rubber composites. As seen in Fig. 3, the SEM image of free silicon rubber, it was found that the silicon rubber cross section morphology was smooth and clear compared to filled composites Fig. 3b–e. In Fig. 3b–d nano tin oxide particles are homogeneous distribution within the silicon rubber sample than micro tin oxide particles, so the nano mixture provided high protection performance^23,24^.

## Mechanical test

The mechanical properties of the sample were tested at room temperature according to ASTM D882-10^21,22^. The investigated samples for tensile properties were manufactured in the form of cuboid of (100 mm _×_ 10 mm _×_ 20 mm) at the test^25–27^.

**Figure 4:**
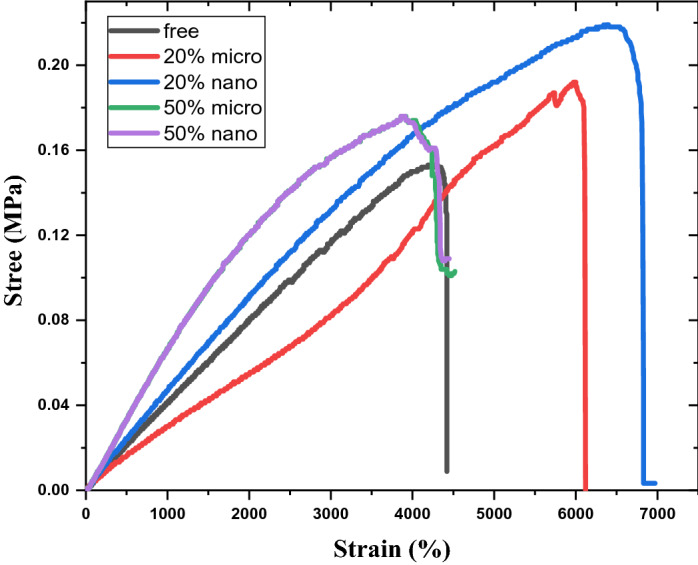
Tensile results of free SR, micro-SnO_2_/SR, and nano-SnO_2_/SR composites.

Figure 4 represents the tensile engineering “stress–strain” characterization curve of free silicon rubber, 20% “nano and micro” SnO_2_/SR, and 50% “nano and micro” SnO_2_/SR composites. The stretching and elongation processes of silicon rubber composites lead to cut-out kinks and high elongation distance “strain” is provided by little stress. Tin oxide filler material improves the elongation distance, tensile stress, and tensile force of silicon rubber composites because of transferring load between tin oxide particles and silicon rubber chains. Also, nano tin oxide particles have good distribution at polymer which led to improved mechanical properties. But, the values of Ultimate tensile stress, Elongation at break, and Ultimate tensile strain of composites decrease by increasing tin oxide concentration from 20 to 50% at micro and nano size. These dramatic changes in tensile properties were due to large agglomerates and aggregates of tin oxide particles which led to decreased SnO_2_/SR composites’ interfacial and cross-linking bonding.

## Radiation measurements

The 30 mm cylinder diameter with 5 mm thickness sample was paced between NaI (TI) cylindrical detector of dimension 3″ × 3″ and the gamma-ray source. A lead collimator with an inner diameter of 8 mm and an outer diameter of 100 mm was used as a house shield for the radioactive source, composite material, and detector. The dead time was less than 3%. The spectrum analyzed using win TMC software. The radioactive sources **(**^241^Am, ^133^Ba, ^60^Co, ^137^Cs and ^152^Eu**)** used were purchased from Physikalisch-Technische Bundesanstalt PTB in Braunschweig and Berlin. The emitted energies are listed in Table 1, which corresponds to the used radioactive source.

**Table 1:** Photon energies, and Half-life time of the radioactive sources used.

Source	Photon Energy (keV)	Half-life time (Days)
Am-241	59.53	157,850
Cs-137	661.66	11,004.98
Co-60	1173.2	1925.31
	1332.5	
Ba-133	80.9	3847.91
	356.01	
Eu-152	121.78	4943.29
	244.69	
	778.9	
	964.1	
	1408.01	

### Radiation parameters

#### Linear attenuation coefficient

The linear attenuation coefficient (μ) is the main parameter to evaluate the effect of the shielding material on the gamma-ray beam, which is calculated by Beer–Lambert low.

1 $$\mu = \frac{1}{x} \ln \frac{I}{{I_{ \circ } }}$$

where, *I* and *I˳* are the transmitted and initial intensities respectively at thickness *x*.

Mass attenuation coefficient is defined as the ratio of the linear attenuation coefficient and material density = $\frac{\mu }{\rho }$^28^.

The relative deviations for the measured mass attenuation coefficient compared to the XCOM result (Dev1) and between micro and nano measured results (Dev2) are given by the following equations:

2 $$Dev1 \%=\frac{XCOM - EXP}{EXP}\mathrm{X }100$$

3 $$Dev2 \%= \frac{Nano - Micro}{Micro} \mathrm{X}100$$

The half and tenth value layer “HVL” and “TVL” are defined as shielding thickness enough to decrease the beam of gamma ray intensity to 50% and 10% of its initial intensity respectively. They are calculated by^29^.

4 $$HVL=\frac{LN (2)}{\mu }$$

5 $$TVL=\frac{LN (10)}{\mu }$$

Effective atomic number (*Z*_*eff*_) parameter is used to describe the shielding properties of composites. It depends on gamma ray energy and pure element properties.

6 $${\text{Z}}_{{{\text{eff}}}} = \frac{{\Sigma_{{i{ }}} w_{i} { }A_{i} { }\left[ {\frac{\mu }{\rho }} \right]_{i} }}{{\Sigma_{{i{ }}} w_{{i{ }}} \frac{{A_{i} }}{{z_{i} }}{ }\left[ {\frac{\mu }{\rho }} \right]_{i} }}$$

where *W*_*i*_, *A*_*i*_ and *Z*_*i*_ are the weight fraction, atomic number and atomic weight of element *i* in composite*, respectively*.

#### Relaxation length ($\uplambda$)

It’s also called mean free path, which is defined as the average distance between two successive interactions between gamma rays and a sample^30^.

7 $${\uplambda } = \frac{1}{{\upmu }}$$

#### The radiation protection efficiency (RPE)

It’s an important parameter used to evaluate the effectiveness of protection material.

8 $$RPE\% = \left[ {1 - \frac{I}{{I_{ \circ } }}} \right] \times 100$$

#### Energy absorption and exposure buildup factors

When a gamma ray beam is incident on a sample, the absorption and scattering interactions between photon and sample depend on photon energy and sample atomic number. Secondary photons result from these interactions, which may be led to an increase in photon flux. Buildup factor is a dimensionless quantity used to evaluate the effect of scattered radiation and deposit secondary photons in the protection process. The equivalent atomic number (Z_eq_) is the first step to calculate the buildup factor which is defined as the ratio between Compton mass attenuation and the total mass attenuation of compounds^30^.

9 $${z}_{eq}=\frac{{z}_{1}(\mathit{log}{R}_{2}-\mathit{log}R)+{z}_{2}(\mathit{log}R-\mathit{log}{R}_{1 })}{(\mathit{log}{R}_{2}-\mathit{log}{R}_{1})}$$

where R_1_ and R_2_ are the ratios of (*μ*_*comp*_*/μ*_*total*_) corresponding to elements which have atomic numbers Z_1_ and Z_2_, respectively. R is the ratio of (*μ*_*comp*_*/μ*_*total*_) corresponding to the composite at defined energy lying between R_1_ and R_2_.

Then, by using Z_eq_ values to get GP fitting parameters (b, c, a, X_k_, d) for free silicon rubber, 20% tin oxide/silicon rubber, and 50% tin oxide/silicon rubber between energy scale from 0.015 MeV to 15 MeV, using the following interpolation equation^31,32^.

10 $$b=\frac{{b}_{1}(\mathit{log}{z}_{2}-\mathit{log}{z}_{eq})+{b}_{2}(\mathit{log}{z}_{eq}-\mathit{log}{z}_{1})}{(\mathit{log}{z}_{2}-\mathit{log}{z}_{1})}$$

**Figure 5:**
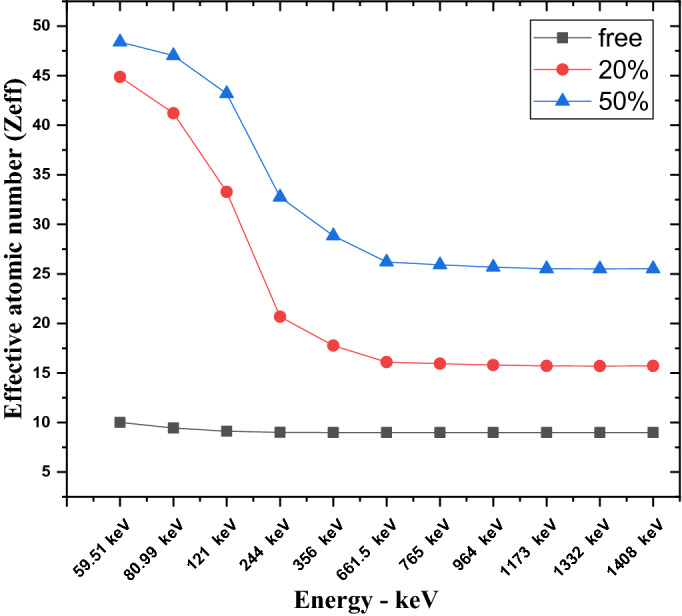
The equivalent atomic number of free silicon rubber, 20% SnO_2_/SR, and 50% SnO_2_/SR for different gamma ray energies.

The curves of the equivalent atomic number Z_eq_ of free silicon rubber, 20% SnO_2_/SR, and 50% SnO_2_/SR against various energies of gamma rays are represented in Fig. 5, where Z_eq_ results depend on Compton effect and it is cross section of interaction. It can be observed that, 50% SnO_2_/SR composite has a higher value than 20% SnO_2_/SR.

Finally, the absorption and exposure buildup factors for selected composite calculated by the computed GP fitting parameters, using following equations.

11 $$B\left(E,x\right)=1+\frac{b-1}{K-1}\left({K}^{x}-1\right) , K\ne 1$$

And

12 $$B\left(E,x\right)=1+\left(b-1\right) x , K=1$$

where

13 $$K\left(E,x\right)=c{x}^{a}+d\frac{\mathrm{tanh}\left(\frac{x}{{X}_{k}}-2\right)-\mathrm{tanh}(-2)}{1-\mathrm{tanh}(-2)} \mathrm{for} x \le 40 \mathrm{mfp}$$

where E is the incident energy at x mean free path, and $K\left(E,x\right)$ is the variation corresponding to the change in energy and spectrum shape^33^.

## Result and discussion

The experimental mass attenuation coefficient (MAC) of free silicon rubber, 20% SnO_2_/SR, and 50% SnO_2_/SR against gamma rays in the range of 60–1408 keV and the corresponding theoretical mass attenuation coefficient obtained from XCOM are listed in Table 2. All obtained experimental results of free silicon rubber, 20% micro SnO_2_/SR, and 50% micro SnO_2_/SR have a good agreement with theoretical XCOM values where the relative deviation (Dev1) results in a range [−3.6 to 4.3] in free silicon rubber, [−2.3 to 1.95] in 20% micro SnO_2_/SR, and [−2.8 to 3.34] in 50% micro SnO_2_/SR. It is observed that, MAC of composites depends on additive “tin oxide” material weight fraction, where MAC decreases with increasing gamma ray and MAC increases gradually with increasing tin oxide percentage. Also, gamma ray energy where at small energies, the MAC decreases gradually because of photoelectric effect, which proportional to “${Z}^{n}$”, *n* = *4–5.* For the medium gamma ray range, MAC dependent on Compton scattering effect “*CE*” which inversely proportional to energy “$CE\propto {E}^{-1}$”. While at higher energies above 1.022 MeV, pair production is the predominant interaction, so that the MAC curve is more alignment in the end of the curve.

**Table 2:** Mass attenuation coefficients, theoretical Xcom, relative deviation, measured linear attenuation coefficient, and measured density, value for free SR, micro-SnO_2_/SR, and nano-SnO_2_/SR composites.

Sample	Energy (keV)	MAC, cm^2^/g					Density (g cm^-3^)	
		Micro			Nano		Micro	Nano
		XCOM	EXP	Dev1 (%)	EXP	Dev2 (%)		
Free silicon rubber	59.51	0.224	0.218	2.690		1.202 ± 0.028		
	80.99	0.184	0.179	2.490				
	121	0.158	0.164	−3.632				
	244	0.156	0.153	2.147				
	356	0.124	0.120	2.331				
	661.5	0.108	0.106	0.877				
	765	0.083	0.079	4.301				
	964	0.078	0.075	3.875				
	1173	0.069	0.067	2.784				
	1332	0.059	0.057	3.821				
	1408	0.057	0.057	−0.052				
20% SnO_2_/SR	59.51	1.184	1.169	1.213	1.428	22.126	1.395 ± 0.006	1.45 ± 0.021
	80.99	0.593	0.607	−2.357	0.752	23.806		
	121	0.281	0.276	1.860	0.336	21.530		
	244	0.136	0.137	−1.127	0.165	20.338		
	356	0.109	0.108	0.770	0.129	19.232		
	661.5	0.081	0.082	−1.681	0.095	15.529		
	765	0.075	0.074	1.868	0.082	10.896		
	964	0.067	0.067	1.049	0.073	9.077		
	1173	0.061	0.061	0.470	0.066	8.180		
	1332	0.057	0.056	1.950	0.060	7.607		
	1408	0.055	0.054	1.816	0.058	6.528		
50% SnO_2_/SR	59.51	2.632	2.588	1.697	3.187	23.159	1.905 ± 0.009	1.967 ± 0.064
	80.99	1.207	1.242	−2.836	1.533	23.470		
	121	0.4701	0.469	0.156	0.571	21.680		
	244	0.155	0.152	1.819	0.184	21.047		
	356	0.112	0.110	2.119	0.128	16.408		
	661.5	0.078	0.078	0.628	0.092	18.294		
	765	0.072	0.070	3.343	0.081	16.345		
	964	0.064	0.065	−1.226	0.075	16.013		
	1173	0.058	0.058	−0.494	0.065	11.826		
	1332	0.054	0.055	−1.236	0.062	13.734		
	1408	0.053	0.054	−1.835	0.060	12.411		

**Figure 6:**
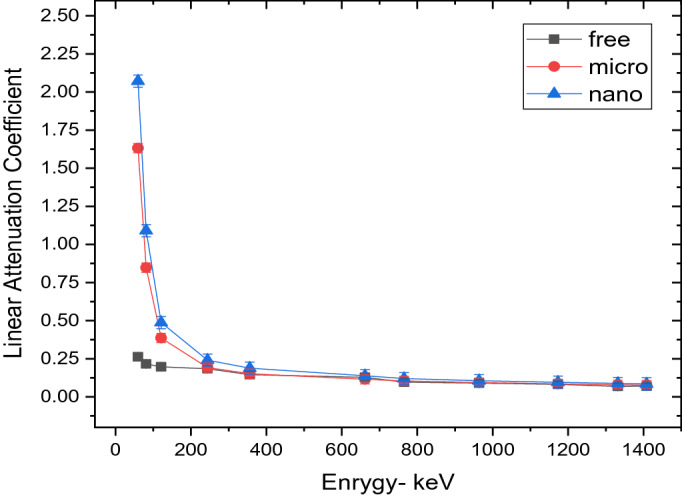
Comparison between LAC for free silicon rubber nano- and micro-SnO_2_ for 20wt% at different energy photon.

**Figure 7:**
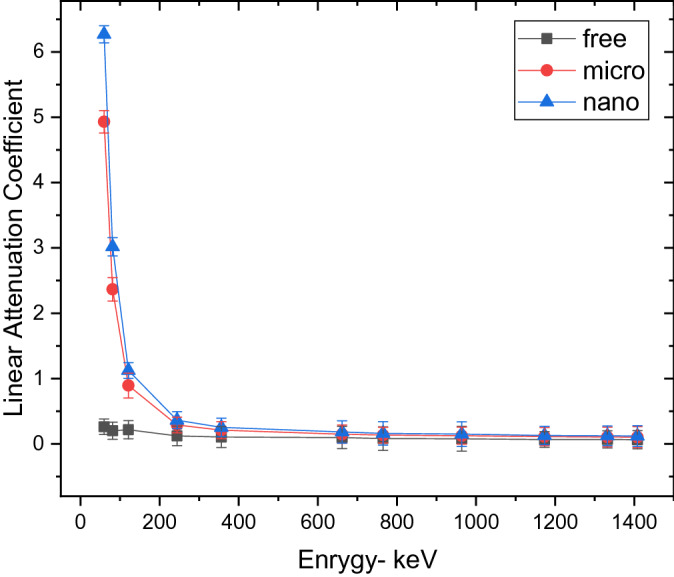
Comparison between LAC for free silicon rubber nano- and micro-SnO_2_ for 50wt% at different energy photon.

**Figure 8:**
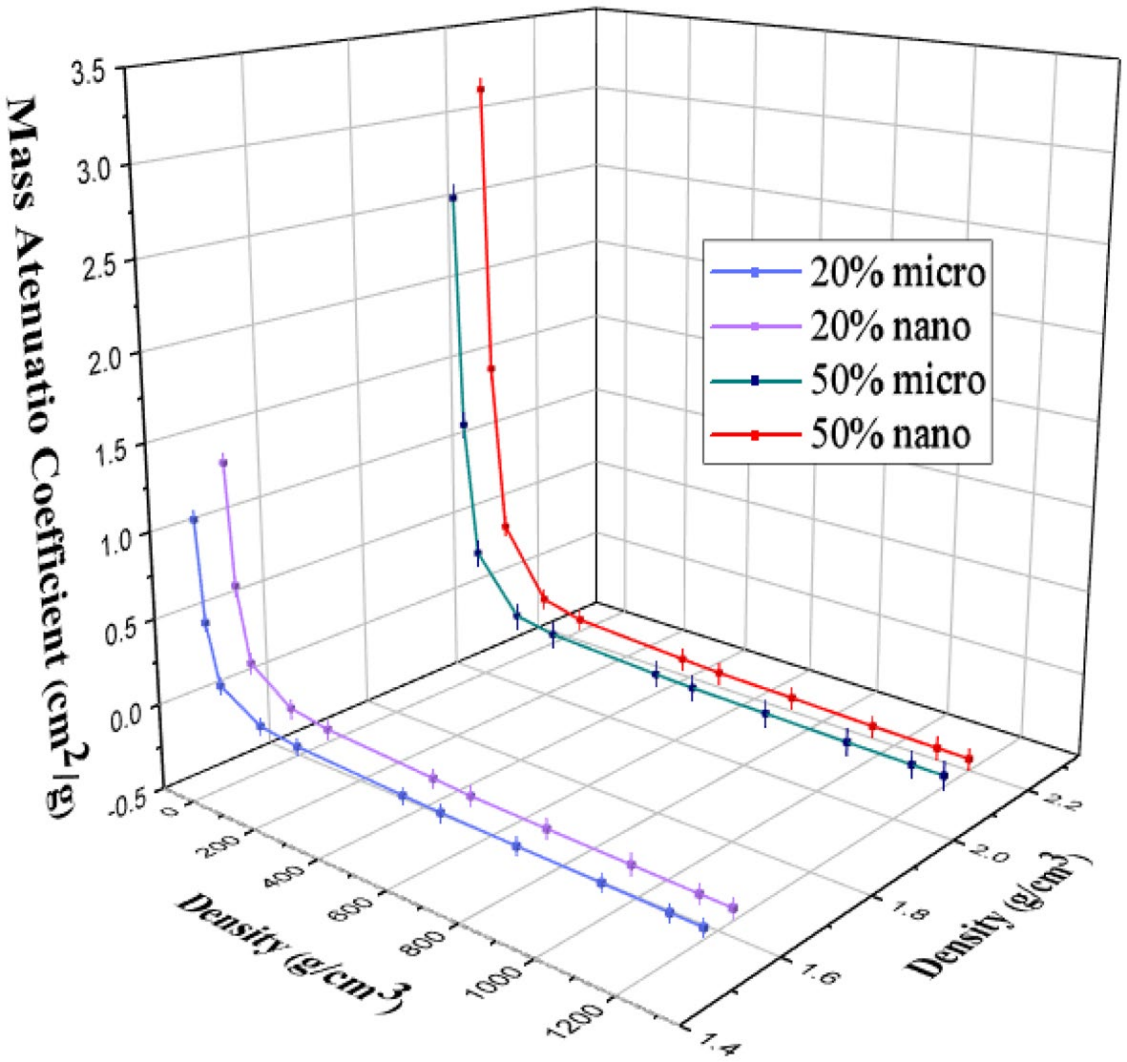
The relationship between the densities of nano- and micro-SnO_2_/SR and their MAC at different energy.

Figures 6 and 7 represent the variation of the linear attenuation coefficient (LAC) of free silicon rubber, 20 and 50% SnO_2_/SR in nano and micro size scale. The nano-sized composites have higher LAC results compared to micro-sized composites. They were observed in both weight fractions, 20 and 50%. The LAC values of 20 and 50% SnO_2_/SR have a highly significant difference between nano and micro composites at low energy, but with increasing energy, the difference between the two curves becomes small and significant. The densities of free silicon rubber, 20% “nano and micro” tin oxide/silicon rubber, and 50% “nano and micro” tin oxide/silicon rubber were measured to evaluate the effectiveness of the weight fraction of additive material and their size scale on the shieling behavior of composites. From Fig. 8, it is observed that, MAC values depend on density for free silicon rubber and SnO_2_/SR at different concentrations and different scales. Figure 8 and Table2 show that, the density of silicon rubber composites increases as the percentage of tin oxide in composites increases, due to their high atomic number and density. Also, composites filled with nano particles have higher density than composites filled with micro at the same weight fraction, which led to increased shielding properties of nano composites than micro composites. There is a high linear correlation coefficient between density and shielding properties such as linear attenuation coefficient.

**Figure 9:**
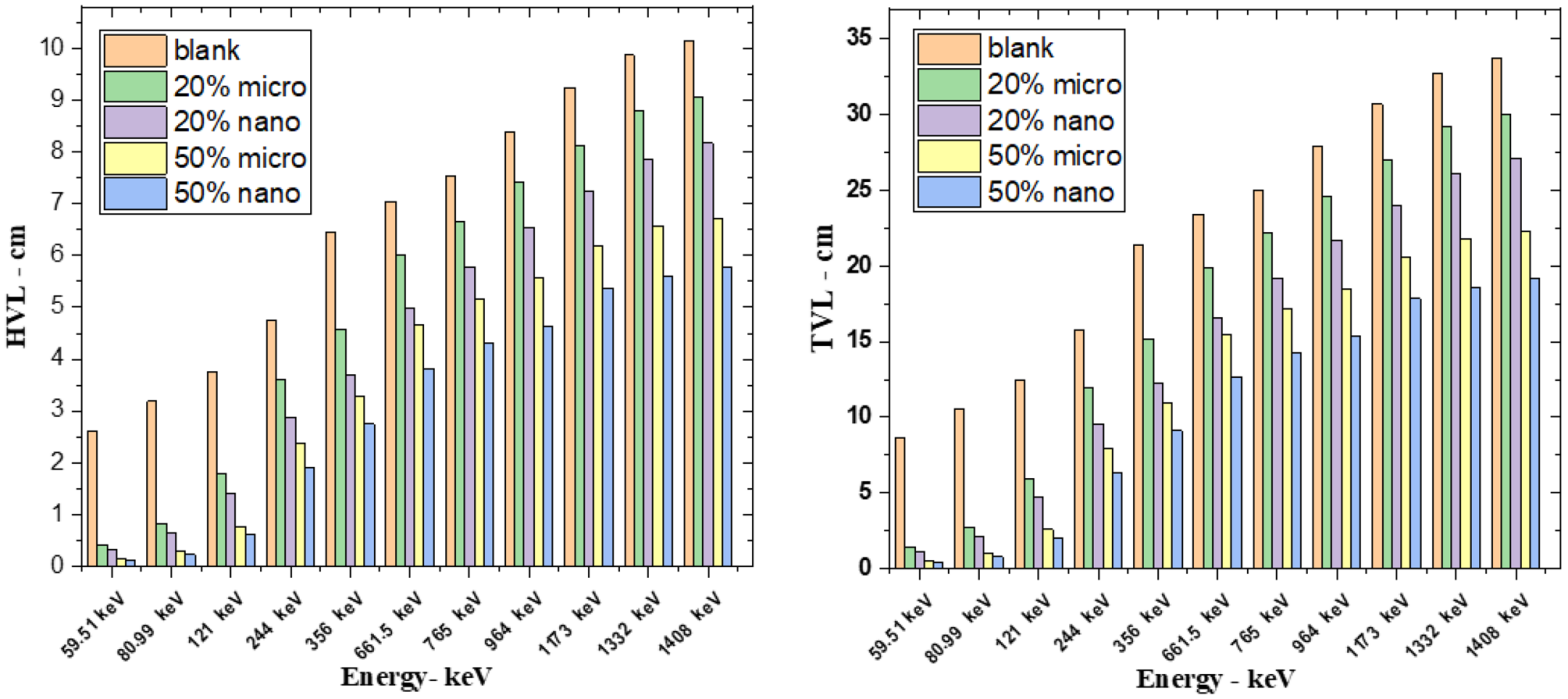
HVL and TVL of free silicon rubber, micro, and nano SnO_2_ at different energy.

The variation results of HVL and TVL are shown in Fig. 9, where HVL and TVL results increase with increasing gamma ray energy gradually at all composites. Free silicon rubber has lower HVL and TVL than the rest of the filled silicon rubber. This explains the reduction and shielding against gamma rays by silicon rubber, which depends on the weight fraction of additive material and its scale size. In other words, the cross section of interaction between photon energy and composites which contain atoms with a high atomic number “SnO_2_” is significantly high and as the percentage of additive material increase, the HVL and TVL values decrease, which means more protection. Nano composites have HVL and TVL results lower than micro composites because nano material has more surface area, leading to more interaction between sample and energy, resulting in more protection.

**Figure 10:**
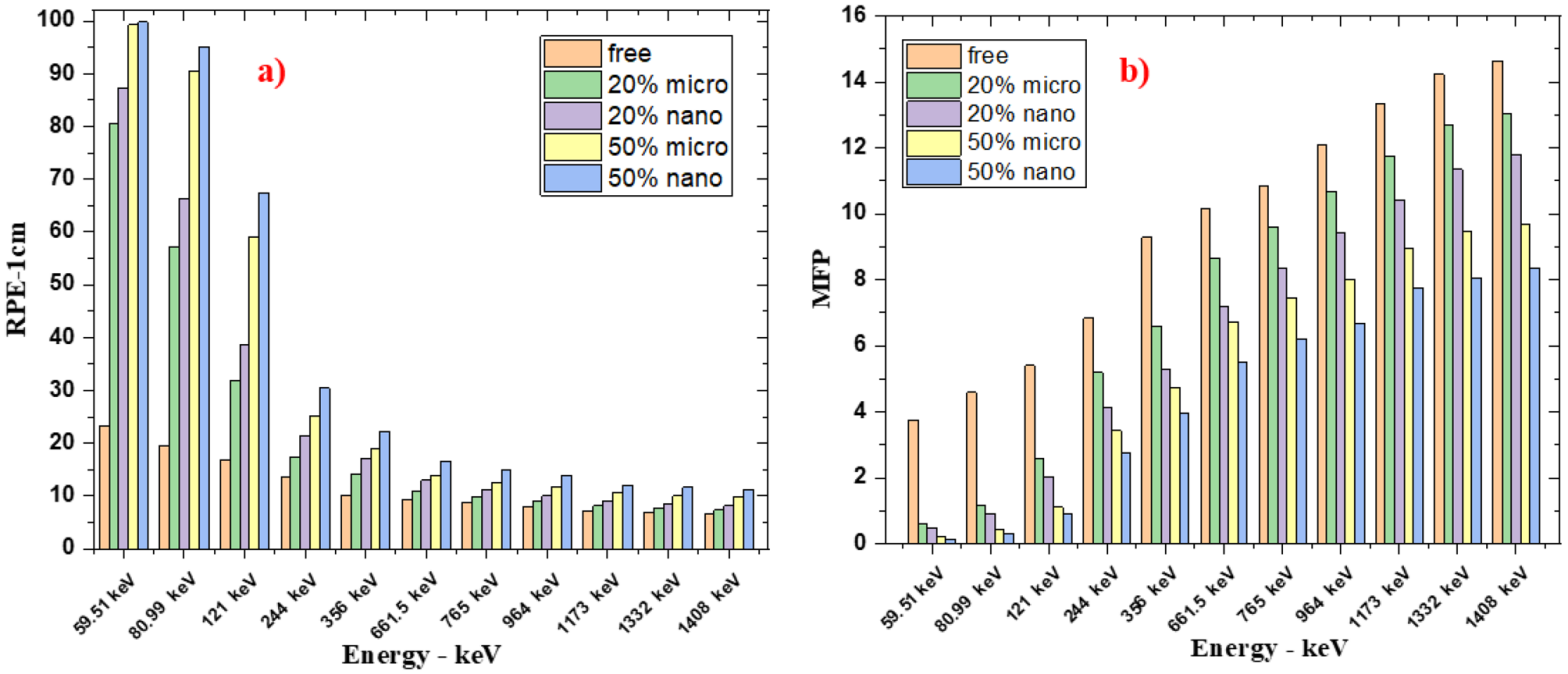
(**a**) Radiation protection efficiency at different energies at 1 cm, (**b**) Mean free path of free silicon rubber and different weight fraction of SnO_2_/SR at different energies.

**Figure 11:**
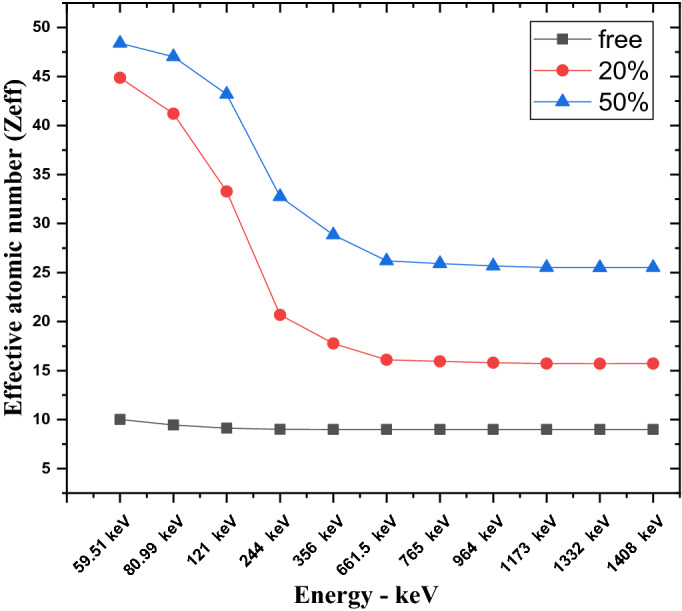
Effective atomic number of free silicon rubber and different weight fraction of SnO_2_/SR at different energies.

The calculated results of RPE at different gamma ray energies for free silicon rubber, 20% “nano and micro” SnO_2_/SR, and 50% “nano and micro” SnO_2_/SR are shown in Fig. 10a. It is observed that, RPE values decrease with increasing photon energy exponentially. 50% nano—SnO_2_/SR has the maximum RPE, it means this composite has the most protection efficiency against gamma rays followed by 50% micro—SnO_2_/SR then 20% nano-SnO_2_/SR then 20% micro- SnO_2_/SR and the last one was free silicon rubber. For more explanation, the existence of additive materials with high Z led to increased interaction between photons and composites and the size scale of material effects on shielding properties. Figure 10b shows the mean free path (MFP) against increasing energy. A lower MFP is preferable because it is the reciprocal of LAC. A lower distance indicates more collisions and increased attenuation because MFP is also the distance between collisions. Figure 11 shows the Z_eff_ results of free silicon rubber, 20% SnO_2_/SR, and 50% SnO_2_/SR for gamma ray energy. It explains the difference in Z_eff_ between the three composites. The Z_eff_ results increase in order free silicon rubber < 20% SnO_2_/SR < 50% SnO_2_/SR. 50% SnO_2_/SR has the maximum Z_eff_ because of presence of tin oxide “SnO_2_” which has the highest atomic number with higher weight fraction than 20% SnO_2_/SR and free silicon rubber. It is observed that Z_eff_ of all composites decreases with an increase of gamma ray energy. The cross section of photon-matter interaction depends on ${Z}^{4-5}/{E}^{3.5}$, therefore in low energy the Z_eff_ has high values for high Z shielding material but in higher energy Z_eff_ of composites energy independent.

**Figure 12:**
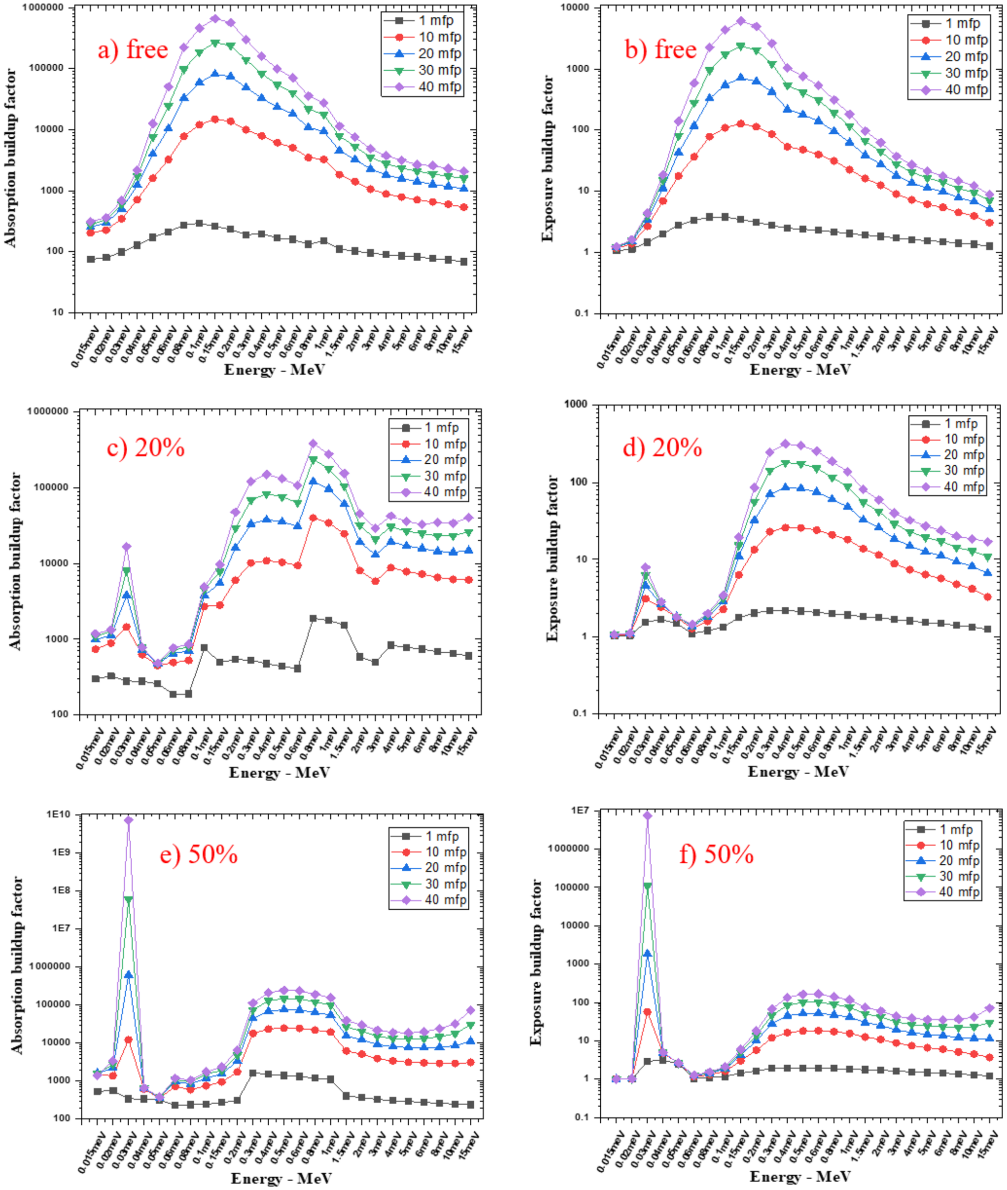
The absorption and exposure buildup factors of free silicon rubber, 20% SnO_2_/SR, and 50% SnO_2_/SR for different energies at 1, 10, 20, 30, and 40mfp.

**Figure 13:**
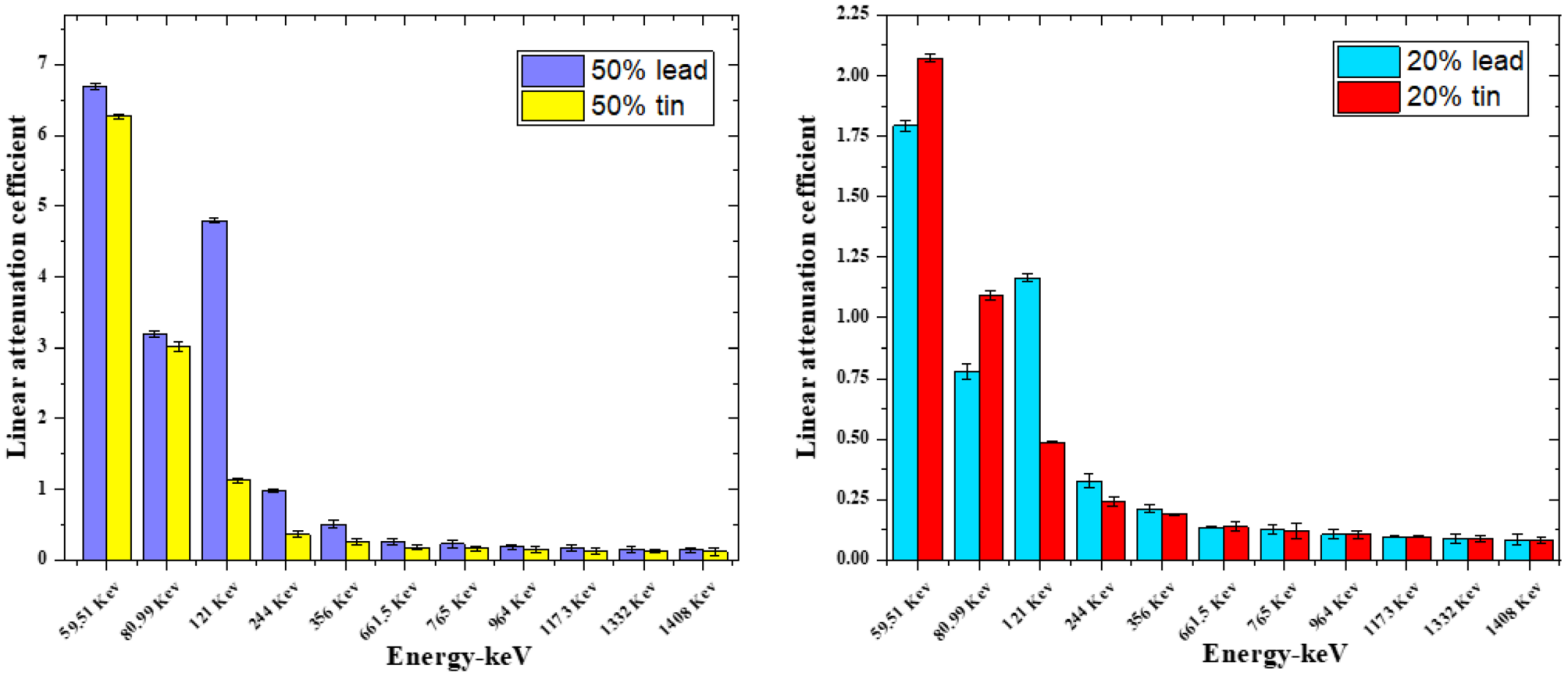
Comparison the LAC between our result and nano-PbO/SR for 20 and 50% concentration at different energy.

From Fig. 12, the buildup factor curves for all composites are low at low energy initially, and as energy increases, the curve increases until it reaches its maximum, then the curve decreases with energy increase. This behavior is because of the predominance of Compton effect, which leads to increased scattering photons in the middle energy range. But, 20% SnO_2_/SR and 50% SnO_2_/SR curves have a peak at 0.030 MeV, which is related to the K-edge of tin, which increases absorption and exposure buildup curves as tin percentage increases, the K-edge peak increases. Figure 13 represent comparison for linear attenuation coefficient at different gamma ray energy between data explained in El-Khatib et al.^34^ where silicon rubber reinforced with nano lead at 20 and 50% weight fraction. Comparison described that LAC of 20% lead was higher than 20%tin in all energy except 59.51 and 80.99 keV only which explained by k-edge of lead in this range of energy. At 50% lead has higher LAC than 50% tin in all energy where densities of lead composites were higher than tin composites in all weight fraction.

## Conclusion

In this work, the radiation protection properties of silicon rubber composites were affected by the particle size and weight fraction of tin oxide, where shielding protection was evaluated by measuring the linear attenuation coefficient and calculating the buildup factor. The composites were prepared by vulcanization technique then their structure characterization explained by SEM imaging and mechanical properties of composites investigated by tensile test. The results of SEM morphology images explain that nanocomposites are more homogenous distributed than micro composites. The experimental results of the mass attenuation coefficient had good agreement with theoretical data from XCOM program. The shielding parameters of nano tin oxide composites are greater than micro tin oxide composites at the same weight fraction and as concentration of tin oxide increases the attenuation parameter increases.

## Data Availability

All data generated or analyzed during this study are included in this published article.
